# Transcriptome profiling reveals superovulation with the gonadotropin-releasing hormone agonist trigger impaired embryo implantation in mice

**DOI:** 10.3389/fendo.2024.1354435

**Published:** 2024-02-26

**Authors:** Meng Li, Jingmei Han, Nana Yang, Xiangyun Li, Xinglong Wu

**Affiliations:** ^1^ College of Animal Science and Technology, Hebei Technology Innovation Center of Cattle and Sheep Embryo, Hebei Agricultural University, Baoding, China; ^2^ College of Veterinary Medicine, Hebei Agricultural University, Baoding, China

**Keywords:** superovulation, gonadotropin-releasing hormone agonist, uterine receptivity, ovary, transcriptome

## Abstract

**Introduction:**

Superovulation is a critical step in assisted reproductive technology, but the use of human chorionic gonadotropin (hCG) as a trigger for superovulation can result in ovarian hyperstimulation. Thus, the use of Gonadotropin-releasing hormone agonist (GnRHa) trigger has been increasingly adopted, although it has been associated with a higher rate of pregnancy failure compared to natural cycles. This study aimed to investigate the effect of GnRHa trigger on embryo implantation in a mouse model.

**Methods:**

Mice in the superovulation (PG) group were administered 7.5 IU of PMSG, followed by the injection of 3.5 μg of GnRHa (Leuprorelin) 48 h later, while mice in the control group (CTR) mated naturally. We compared the number of oocytes, blastocysts, and corpus luteum between the two groups and the implantation sites after the transfer of natural blastocysts. Ovaries, uterus, and serum 2 and 4 days after mating were collected for qRT-PCR, transcriptome sequencing, and hormone assays.

**Results:**

The PG group had more oocytes, blastocysts, and corpus luteum after superovulation than the CTR group. However, the mRNA expression of leukemia inhibitory factor (*Lif*) and the number of implantation sites were reduced in the PG group. The ELISA assay revealed that superovulation increased ovarian estrogen secretion. The transcriptome analysis showed that superphysiological estrogen led to a response of the uterus to a high estrogen signal, resulting in abnormal endometrium and extracellular matrix remodeling and up-regulation of ion transport and inflammation-related genes.

**Conclusion:**

Our findings suggest that a combination of PMSG and GnRHa trigger impaired embryo implantation in mice, as the excessive uterine response to superphysiological estrogen levels can lead to the change of gene expression related to endometrial remodeling, abnormal expression of uterine ion transport genes and excessive immune-related genes.

## 1 Introduction

Embryo implantation is a highly coordinated maternal-embryonic communication process (1). Successful embryo implantation requires an implantable blastocyst and a receptive uterus. The uterus undergoes dramatic changes to receive a mature blastocyst, including significant molecular changes and tissue remodeling, such as the proliferation inhibition of endometrial epithelial cells and the decidualization of stromal cells (2). These changes are mainly driven by estrogen and progesterone (2, 3). Subtle hormonal changes during implantation can significantly impact the environment of the uterus. Superovulation technology is widely used in human-assisted and large-scale animal reproduction. In humans, superovulation is an essential step within *in vitro* fertilization-embryo transfer (IVF-ET) cycles and is a popular technology to obtain many oocytes (4, 5). In superovulation protocols, human chorionic gonadotrophin (hCG) is commonly used to trigger ovulation (6). However, hCG has been shown to cause ovarian hyperstimulation and produce stunted embryos (7, 8). The half-life of hCG is as long as 24 h, which can lead to significant changes in the hormonal environment (9). This nonphysiological maternal hormonal environment continues through embryo implantation and early placenta formation (10, 11). Gonadotropin-releasing hormone agonists (GnRHa), which have a shorter half-life as an alternative to hCG, reduce the occurrence of ovarian hyperstimulation (7, 9, 12). At present, GnRHa has been gradually used to replace hCG to trigger ovulation in human-assisted reproductive technology. However, the stimulation of the ovaries with GnRHa still results in more pregnancy failures compared to the natural cycle (13, 14). Such results can be related to abnormal hormone levels caused by impaired ovarian function after superovulation.

The classic superovulation protocol of the mouse model administers hCG to induce ovulation after the intraperitoneal injection of pregnant mare serum gonadotropin (PMSG) for 48 h (15). In the mouse model, the GnRHa trigger results in ovulation like the hCG trigger (16, 17). Decreased VEGF expression in the ovaries was observed with the GnRHa trigger compared to the hCG trigger, which could explain the reduced ovarian hyperstimulation. Numerous research works have evaluated the ovarian, uterine, and fetal abnormalities caused by superovulation (18–20). Previous studies have shown that superovulation with the hCG trigger alters the expression of genes about tissue remodeling and placenta formation during implantation, resulting in abnormal placental and fetal growth (10, 21). The study found that GnRHa triggers altered the expression of angiogenic factors in the mouse uterus (17). Another study found that after the GnRHa trigger, the mouse uterus showed abnormal leukocyte distribution and higher inflammatory response (22). There are several studies showed that the use of HCG to trigger ovulation leads to impaired uterine receptivity (20, 23). However, the effect of GnRHa-triggered protocols on embryo implantation has not been clarified, especially the ovarian and uterine changes in pre-implantation.

In this study, we used natural blastocyst transfer into the uterus of mice, which used the GnRHa trigger to explore the effect of the GnRHa trigger on uterine receptivity. In mice, the maximal receptive period of the uterus for the embryo (the implantation window) typically occurs 4.5-5 days post coitus (dpc) (2). Therefore, the uterus and ovaries were collected on 2 dpc (before implantation) and 4 dpc (close to implantation), and then transcriptome sequencing was performed, respectively. The results revealed the impact of the superovulation protocol of PMSG combined with GnRHa on the transcriptome expression profile of the ovary and uterus before implantation. This study provides valuable insights for optimizing the outcome of assisted reproduction.

## 2 Materials and methods

### 2.1 Animals

All mice (CD-1, 6-8 weeks old, 20-25 g) were purchased from Beijing Vital River Laboratory Animal Technology Co., Ltd. (Beijing, China). All mice were housed at 20-25°C with 12 h/12 h light-dark cycles and received food and water ad libitum during the experiment. Male mice that received a vasectomy were used to create pseudopregnant mice. The Ethical Committee of Hebei Agricultural University approved this study.

### 2.2 Superovulation, embryo transfer, and samples collection

102 female mice were split randomly into two groups of 51 mice each. For the superovulation (PMSG combined with GnRHa, PG) group, mice were administered 7.5 IU of PMSG (Ningbo Sansheng Biological Technology Co., Ltd) by an Intra-peritoneal (i.p) injection, followed by an i.p injection of 3.5 μg of GnRHa (Leuprorelin, Selleckchem) 48 h later. At the same time, the female mice mated to males. The control (CTR) group mice were not treated with hormones and mated with males during estrus. Vaginal plugs confirmed successfully mating the following day when it was designated 1-day post coitum (dpc). Cumulus–oocyte complexes were collected in the oviduct on 1 dpc morning (no mating was required in the PG group), and cumulus cells were then removed in the 0.1% hyaluronidase. Blastocysts were collected at the uterine horn on 4 dpc mornings. The number and quality of oocytes and blastocysts were counted. In embryo transfer experiments, recipients mated with vasectomy males. Natural blastocysts (7 or 8) were transferred into a single uterine horn of the recipients on 3 dpc. Embryo implantation was examined on 8 dpc. Furthermore, we collected the ovary, uterine horn, and serum of pseudopregnant mice on 2 dpc mornings and 4 dpc nights. One side of the ovary of three mice was stored in 4% paraformaldehyde, and the other was stored in an RNAlater™ solution (Invitrogen) at -20°C until RNA extraction. The uterine was stored in an RNAlater™ solution (Invitrogen) at -20°C until RNA extraction. Mice blood was collected from a retro-orbital vein and centrifuged at 3000 rpm for 10 min at 4°C to extract serum. The serum was stored at -20°C until the enzyme-linked immunosorbent assay (ELISA) was used. In the reproduction experiment, eight mice in the CTR and PG groups were selected to give birth, and the number of pups in each group was counted.

### 2.3 Haematoxylin-eosin staining of the ovaries

The ovaries were fixed in 4% paraformaldehyde for more than 48 h. Ovaries were placed in embedding boxes and rinsed under running water for 12 h. After dehydration with an alcohol gradient, the ovaries were immersed in xylene for 15 min, soft wax for 60 min, and hard wax for 60 min, and each step was repeated twice. The tissue was embedded and cut into 5 μm sections in an embedding machine, and the slices were dried at 50°C for more than 30 min before being deparaffinized and rehydrated. HE staining was performed on these sections. The ovarian tissue sections were observed under a microscope, and the number of corpus luteum was counted.

### 2.4 Enzyme-linked immunosorbent assay

Serum samples were analyzed using an enzyme-linked immunosorbent assay (ELISA) kit (Shanghai Jianglai Biotechnology Co., Ltd., China). The E_2_ ELISA kit on the intra-assay CV was less than 9%, the inter-assay CV was less than 11%, and the assay sensitivity was 0.1 pg/mL. The P_4_ ELISA kit on the intra-assay CV was less than 9%, the inter-assay CV was less than 11%, and the assay sensitivity was 0.1 ng/mL.

### 2.5 RNA extraction, library construction, and sequencing

The total RNA was extracted using a Trizol reagent kit (Invitrogen) according to the manufacturer’s protocol. RNA quality was assessed on an Agilent 2100 Bioanalyzer (Agilent Technologies, Palo Alto, CA, USA) and RNase-free agarose gel electrophoresis. After the total RNA was extracted, mRNA was enriched by Oligo(dT) beads. Then, the enriched mRNA was fragmented into short fragments using a fragmentation buffer and reversely transcribed into cDNA using the NEBNext Ultra RNA Library Prep Kit for Illumina (NEB). The purified cDNA fragments were end-repaired, and the A base was added and ligated to Illumina sequencing adapters. The ligation reaction was purified with AMPure XP Beads (1.0X). Ligated fragments were subjected to size selection by agarose gel electrophoresis and a polymerase chain reaction (PCR), which was amplified to obtain the final cDNA library. The cDNA library was sequenced using Illumina No-vaseq6000 by Gene Denovo Biotechnology Co. (Guangzhou, China).

### 2.6 Quality control of sequencing data

The reads were further filtered by using fastp (version 0.18.0). The parameters were as follows: 1) removing reads containing adapters; 2) removing reads containing more than 10% of the unknown nucleotides(N); 3) removing low-quality reads containing more than 50% of low-quality (Q-value ≤ 20) bases. The short read alignment tool Bowtie2 (version 2.2.8) was used for mapping reads to the ribosome RNA (rRNA) database. The rRNA-mapped reads were then removed. The remaining clean reads were further used in an assembly and gene abundance calculation.

### 2.7 Quantification of gene expression level

The clean reads were mapped to the reference genome using HISAT2, and then mapped reads were assembled using StringTie v1.3.1. The FPKM (fragment per kilobase of transcript per million mapped reads) value was calculated for each transcription region to quantify its expression abundance and variations using RSEM software. RNA differential expression analysis was performed by DESeq2 software between the two groups (and by edgeR between two samples). The genes with the parameter of a false discovery rate (FDR) < 0.05 and absolute fold change > 2 were considered differentially expressed genes (DEGs).

### 2.8 Gene ontology and pathway enrichment analyses

All DEGs were mapped to GO terms in the Gene Ontology database (http://www.geneontology.org/), gene numbers were calculated for every term, and significantly enriched GO terms in DEGs compared to the genome background were defined by a hypergeometric test (*p* < 0.05). The Kyoto Encyclopedia of Genes and Genomes (KEGG) Pathway enrichment analysis identified significantly enriched metabolic pathways or signal transduction pathways in DEGs compared with the whole genome background (*p* < 0.05).

### 2.9 Quantitative real-time PCR

The total RNA was extracted from the tissues using the RNeasy Plus Mini Kit (QIAGEN, Germany), and the total RNA was reverse transcribed into cDNA as required by the PrimeScript RT reagent kit (TaKaRa, Japan). SYBR fluorescent dye (Biotium, USA) was used for qRT-PCR. The reaction system was 2× qPCR Master Mix 10 μL, the forward primer was 0.4 μL, the reverse primer was 0.4 μL, and cDNA 1 μL, rox 3 μL, and ddH2O were supplemented to 20 μL. The reaction conditions were as follows: predenaturation at 95°C for 120 s, denaturation at 95°C for 15 s, 60°C annealing for 30 s, and amplification for 40 cycles. The 2^-△△Ct^ method was used to analyze data. Primers were synthesized by Sangon Biotech (Shanghai, China). The primer information for qRT-PCR is provided in **Table 1**.

**Table 1:** Primer sequences used for qRT–PCR.

Gene	Primer sequences (5’-3’)	Accession No.
*Gapdh*	Forward: AGGTCGGTGTGAACGGATTTG Reverse: TGTAGACCATGTAGTTGAGGTCA	NM_001289726.2
*Lif*	Forward: ATTGTGCCCTTACTGCTGCTG Reverse: GCCAGTTGATTCTTGATCTGGT	NM_001039537.3
*Hoxa10*	Forward: CCTGCCGCGAACTCCTTTT Reverse: GGCGCTTCATTACGCTTGC	NM_008263.4
*Itgb3*	Forward: CCACACGAGGCGTGAACTC Reverse: CTTCAGGTTACATCGGGGTGA	NM_016780.2
*Inhba*	Forward: AAATCAGAACGCCTCCGCTA Reverse: TCCCGAGTGTAGAGTTCGGT	NM_008380.2
*Cyp17a1*	Forward: TGGAGGCCACTATCCGAGAA Reverse: CACATGTGTGTCCTTCGGGA	NM_007809.3
*Hsd17b7*	Forward: ATAATGTGGCTCGTGTGGCT Reverse: ATGTCCATCTTTTGGCCCGT	NM_001420237.1
*Prap1*	Forward: AGAAGGTCTGGGATACTAGAGCC Reverse: GCATCTGGACGCTTTTCCTC	NM_009475.2
*H2-Ea*	Forward: CGTCTGAGGCTACCCCTTTC Reverse: GAGAACCCCAGCCAGACATT	NM_010381.3

### 2.10 Statistical analysis

The data were analyzed for significance using unpaired T-test except for sequencing data, and the results are expressed as the data mean ± SEM. *p* < 0.05 was considered statistically significant. Sequencing data are expressed as FPKM mean ± SEM. Images were produced using GraphPad Prism 8.0 software and Omicsmart (www.omicsmart.com).

## 3 Results

### 3.1 Superovulation impaired uterine receptivity in mice

**Figure 1:**
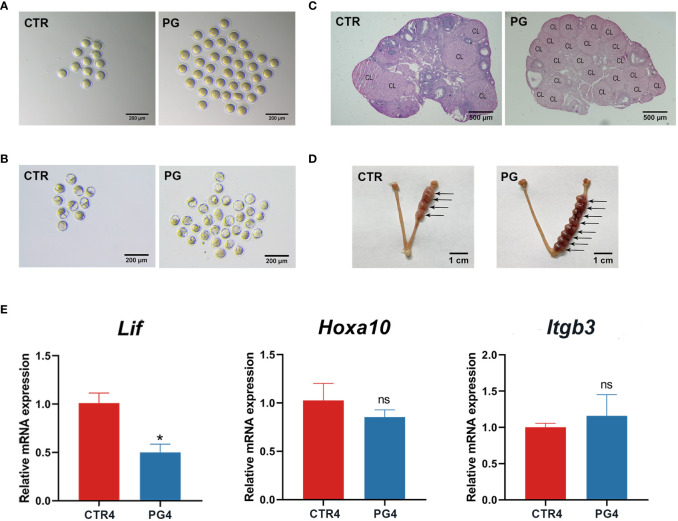
Superovulation led to impaired implantation in mice. **(A)** The difference of ovulations in control (CTR) and superovulation (PG) groups on 1 dpc. (n = 7). **(B)** The difference of blastocysts in CTR and PG groups on 4 dpc. (n = 7). **(C)** The difference of corpus luteum (CL) in CTR and PG groups on 4 dpc. (n = 3). **(D)** Implantation sites in CTR and PG groups on 8 dpc. (n = 29). **(E)** Uterine receptivity related gene mRNA expression in CTR and PG groups on 4 dpc. (n = 3). *Lif*, leukemia inhibitory factor. *Hoxa10*, homeobox A10. *Itgb3*, integrin beta 3. CTR4, control group on 4 dpc. PG4, superovulation group on 4 dpc. *p < 0.05. ns, no significance. Scale bar: **(A, B)** is 200 µm; **(C)** is 500 µm; **(D)** is 1 cm.

After administering PMSG combined with GnRHa for superovulation, we found that the number of recovered oocytes in the PG group was significantly higher than in the CTR group (**Table 2**, **Figure 1A**). Although there were a few poorly developed embryos in the PG group, there were more blastocysts in the PG group than in the CTR group (**Table 2**, **Figure 1B**). At the same time, there was a higher number of corpus luteum in the ovary of the PG group compared to the CTR group on 4 dpc (**Table 2**, **Figure 1C**). The reproductive test results indicated that although the PG group had more pups than the CTR group, there was a discrepancy in the number of blastocysts of the PG group (**Table 3**). We used embryo transfer on mice to eliminate the effect of embryo number and quality on the implantation rate. 7-8 natural embryos were transferred to one side of the uterine horn of each recipient mouse. Compared with the CTR group, the PG group exhibited a significant decrease in the implantation rate (70.09% vs. 24.47%, **Table 4**) and fewer implantation sites (5.66 vs. 2.03, **Table 4**, **Figure 1D**). These findings indicate that most pre-implantation embryos appear capable of developing into blastocysts following superovulation, and the reduction in implantation sites is mainly affected by the uterine environment.

**Table 2:** Statistics of the corpus luteum, oocyte, and embryo number in mice.

Group	Females (No.)	Oocytes (No.)	Oocytes per female (No.)	Blastocysts (No.)	Blastocysts per female (No.)	Corpus luteum (No.)
**CTR**	7	83	11.86 ± 2.23	79	11.29 ± 0.70	5.57 ± 0.34
**PG**	7	215	30.71 ± 8.55**	201	28.71 ± 8.71**	18.57 ± 0.78**

Values are mean ± SEM. CTR, control group. PG, superovulation (PMSG combined with GnRHa, PG) group. **p < 0.01.

**Table 3:** Statistics of pups number.

Group	Females (No.)	Pups (No.)	Pups per female (No.)
**CTR**	8	88	11 ± 1.05
**PG**	8	118	14.75 ± 2.48^ns^

Values are mean ± SEM. CTR, control group. PG, superovulation (PMSG combined with GnRHa, PG) group. ns, no significance.

**Table 4:** Statistics of embryo implantation rates by embryo transfer in mice.

Group	Recipients (No.)	Embryos (No.)	Implantation sites (No.)	Implantation sites per female (No.)	Implantation rate (%)
**CTR**	29	234	164	5.66 ± 1.89	70.09 ± 4.53
**PG**	29	282	69	2.03 ± 2.08**	24.47 ± 4.65**

Values are mean ± SEM. CTR, control group. PG, superovulation (PMSG combined with GnRHa, PG) group. **p < 0.01.

On 4 dpc, we used qRT-PCR to evaluate the uterine receptivity-related genes, including the leukemia inhibitory factor (*Lif*), homeobox A10 (*Hoxa10*), and integrin beta 3 (*Itgb3*). The results showed that *Lif* expression was significantly decreased, and the *Hoxa10* and *Itgb3* expression showed no significant difference (**Figure 1E**). These results suggested that superovulation affects uterine receptivity and leads to embryo implantation failure.

### 3.2 Transcriptome sequencing data quality control and differentially expressed genes identification

We performed quality control on the raw data before analysis to reduce the analysis interference caused by invalid data. We acquired between 10.9 and 30.9 million clean reads per sample after filtering the fastq files. We used bowtie2 to map the clean reads to the ribosome database, removed the mapped reads, and used unmapped reads for transcriptome analysis. After mapping the unmapped reads to the reference genome, we used Stringtie to reconstruct the transcripts, obtained the expression levels of all genes in each sample, and corrected them to FPKM values for subsequent analysis.

**Figure 2:**
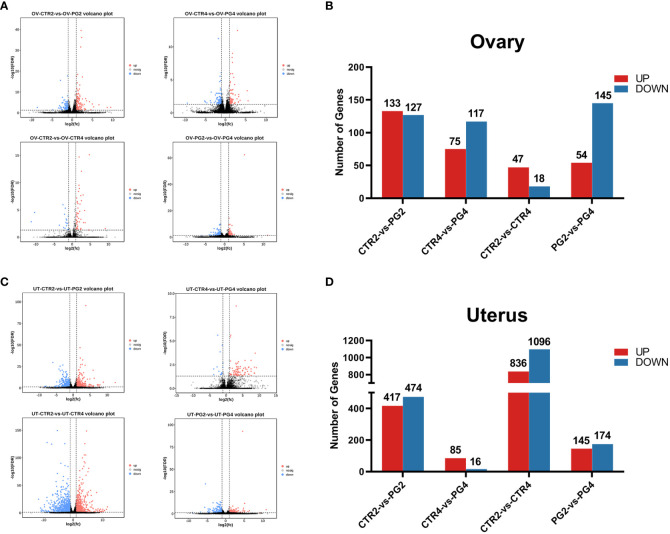
Transcriptome differences in mice ovary and uterus by superovulation and different time points after mating. **(A, B)** Volcano plots and bar plots of differentially expressed genes (DEGs) in ovaries between different groups. **(C, D)** Volcano plots and bar plots of differentially expressed genes in the uterus between different groups. CTR2, control group on 2 dpc. PG2, superovulation group on 2 dpc. CTR4, control group on 4 dpc. PG4, superovulation group on 4 dpc. (n = 3).

We assessed the differences in gene expression caused by superovulation in the ovaries and uterus. The genes with FDR < 0.05 and a fold change > 2 were selected as differential genes. On 2 dpc, superovulation resulted in 260 (133 up-regulated, 127 down-regulated) differential expression genes (DEGs) in the ovary and 891 DEGs (417 up-regulated, 474 down-regulated) in the uterus compared with the CTR group. On 4 dpc, compared with the CTR group, superovulation resulted in 192 DEGs in the ovary (75 up-regulated, 117 down-regulated) and 101 DEGs (85 up-regulated, 16 down-regulated) in the uterus. From 2 dpc to 4 dpc, In the CTR group, there were 65 DEGs (47 up-regulated, 18 down-regulated) and 1932 genes (836 up-regulated, 1096 down-regulated) that showed differential expression in the ovary and uterus, respectively. In the PG group, 199 (54 up-regulated, 145 down-regulated) and 319 genes (145 up-regulated, 174 down-regulated) were differentially expressed in the ovary and uterus, respectively. (**Figures 2A–D**).

### 3.3 Superovulation perturbed ovarian hormone secretion and uterus respond to high estrogen signals

**Figure 3:**
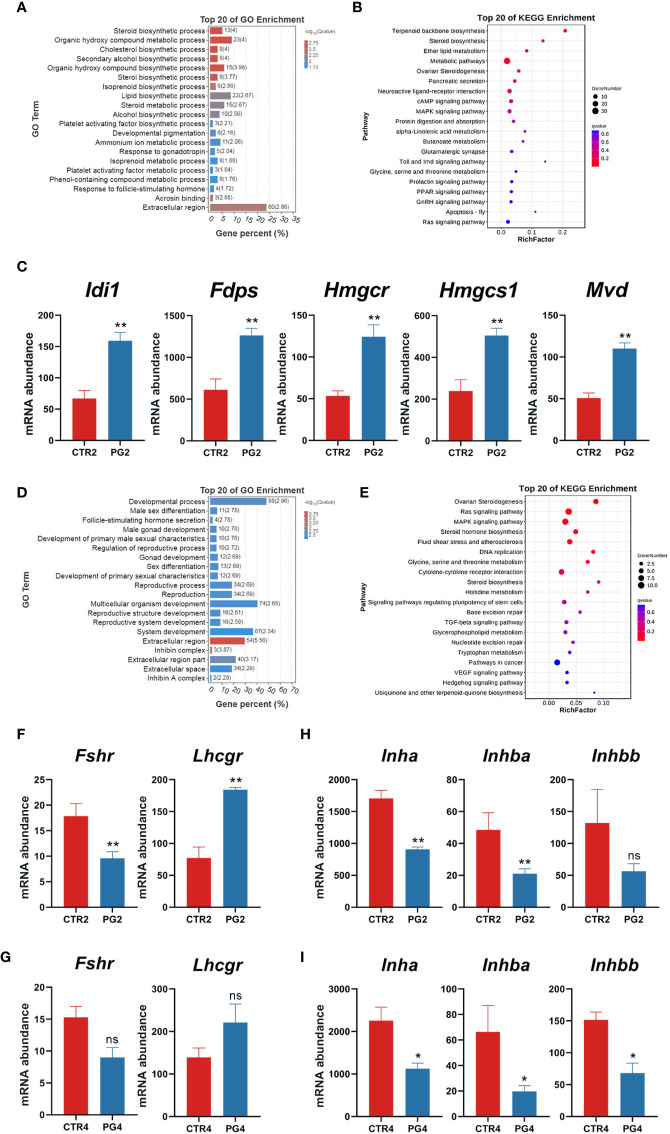
Superovulation caused the abnormal ovarian response to gonadotropins. **(A)** The top 20 of Gene Ontology (GO) analysis of differentially expressed genes (DEGs) between CTR2 and PG2 groups. **(B)** The top 20 of Kyoto Encyclopedia of Genes and Genomes (KEGG) pathway analysis of DEGs between CTR2 and PG2 groups. **(C)** DEGs enriched in terpenoid backbone biosynthesis between CTR2 and PG2 groups. **(D)** The top 20 of GO analysis of DEGs between CTR4 and PG4 groups. **(E)** The top 20 of KEGG pathway analysis of DEGs between CTR4 and PG4 groups. **(F)** Follicle-stimulating hormone receptor (*Fshr*) and luteinizing hormone/choriogonadotropin receptor (*Lhcgr*) mRNA expression between CTR2 and PG2 groups. **(G)** *Fshr* and *Lhcgr* mRNA expression between CTR4 and PG4 groups. **(H)** Inhibin-related gene mRNA expression between CTR2 and PG2 groups. **(I)** Inhibin-related gene mRNA expression between CTR4 and PG4 groups. *Idi1*, isopentenyl-diphosphate delta isomerase. *Mvd*, mevalonate (diphospho) decarboxylase. *Hmgcs1*, 3-hydroxy-3-methylglutaryl-Coenzyme A synthase 1. *Hmgcr*, 3-hydroxy-3-methylglutaryl-Coenzyme A reductase. *Fdps*, farnesyl diphosphate synthetase. *Inha*, inhibin alpha. *Inhba*, inhibin beta-A. *Inhbb*, inhibin beta-B. CTR2, control group on 2 dpc. PG2, superovulation group on 2 dpc. CTR4, control group on 4 dpc. PG4, superovulation group on 4 dpc. *FDR < 0.05. **FDR < 0.01. ns, no significance. (n = 3).

To further evaluate the effect of superovulation on embryo implantation, we performed the GO and KEGG enrichment analysis of DEGs in the ovary. On 2 dpc, DEGs were enriched in biological process terms in relation to ovarian steroid hormones, such as the steroid biological process, cholesterol biological process, and steroid metabolic process (**Figure 3A**). The KEGG pathway analysis showed that DEGs were significantly enriched in terpenoid backbone biosynthesis (**Figure 3B**). The analysis of DEGs enriched in this term showed that all the genes abundance were significantly increased (**Figure 3C**). These genes were also enriched in steroid biosynthesis. On 4 dpc, DEGs are enriched in the FSH secretion process about inhibin in the cell components, molecular functions, and biological processes (**Figure 3D**). Among the DEGs, the abundance of *Fshr* was down-regulated, and the abundance of *Lhcgr* was up-regulated in the ovary on 2 dpc (**Figure 3F**). In contrast, the abundance of inhibin-related genes (*Inha*, *Inhba*) was down-regulated (**Figure 3H**). On 4 dpc, *Fshr* (FDR = 0.164, *p* = 0.012) and *Lhcgr* (FDR = 0.164, *p* = 0.017) had the same expression trend as on 2 dpc (**Figure 3G**), but the difference was not significant. Similarly, the abundance of inhibin-related genes (*Inha*, *Inhba*, *Inhbb*) was down-regulated on 4 dpc (**Figure 3I**). We speculated that the continuous stimulation of the pituitary gland by GnRHa could reduce the expression of inhibin in the ovary. These led to the excessive secretion of FSH and the induced LHCGR expression in granulosa cells, affecting ovarian function. DEGs were significantly enriched in Ovarian Steroidogenesis, the Ras signaling pathway, and the MAPK signaling pathway by KEGG analysis (**Figure 3E**). The enriched Ras signaling pathway and the MAPK signaling pathway were related to cell proliferation, differentiation, and apoptosis.

**Figure 4:**
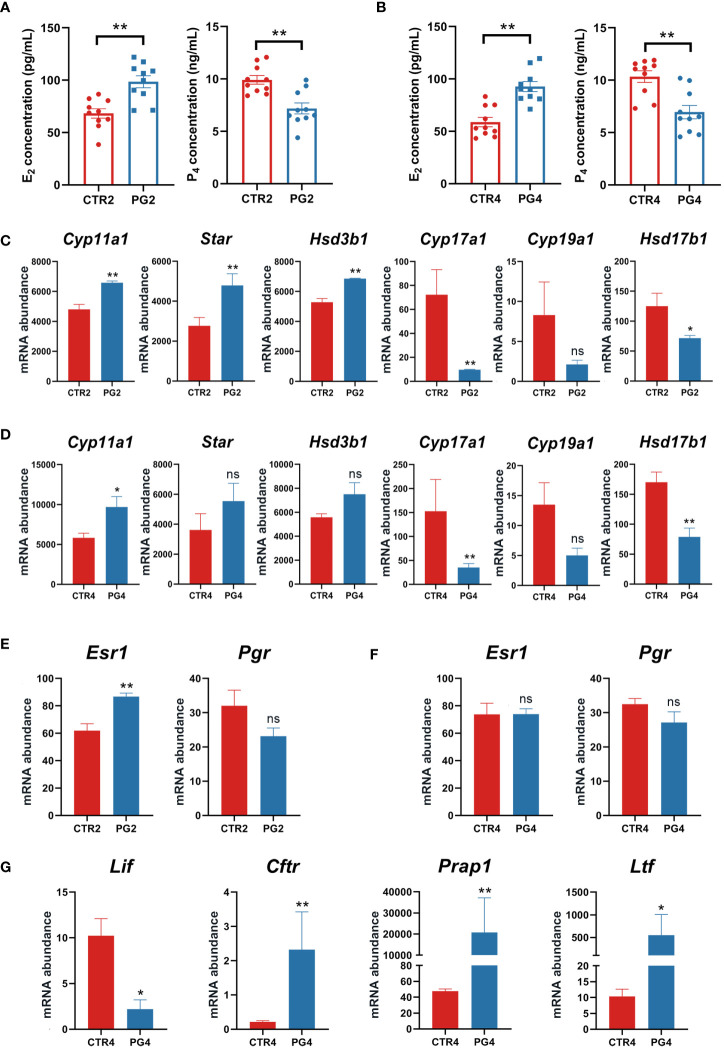
Superovulation led to abnormal ovarian steroid hormone synthesis and secretion. **(A)** Estrogen (E_2_) and progesterone (P_4_) concentrations between CTR2 and PG2 groups. **(B)** Estrogen (E_2_) and progesterone (P_4_) concentrations between CTR4 and PG4 groups. **(C)** The mRNA expression of key enzymes for steroid hormone synthesis between CTR2 and PG2 groups in the ovary. **(D)** The mRNA expression of key enzymes for steroid hormone synthesis between CTR4 and PG4 groups in the ovary. **(E)** Estrogen receptor 1 (*Esr1*) and progesterone receptor (*Pgr*) mRNA expression between CTR2 and PG2 groups in the uterus. **(F)** *Esr1* and *Pgr* mRNA expression between CTR4 and PG4 groups in the uterus. **(G)** DEGs of E_2_-responsive gene mRNA expression between CTR4 and PG4 groups in the uterus. *Cyp11a1*, cytochrome P450, family 11, subfamily a, polypeptide 1. *Star*, steroidogenic acute regulatory protein. *Hsd3b1*, hydroxy-delta-5-steroid dehydrogenase, 3 beta and steroid deltaisomerase 1. *Cyp17a1*, cytochrome P450, family 17, subfamily a, polypeptide 1. *Cyp19a1*, cytochrome P450, family 19, subfamily a, polypeptide 1. *Hsd17b1*, hydroxysteroid (17-beta) dehydrogenase 1. *Lif*, leukemia inhibitory factor. *Cftr*, cystic fibrosis transmembrane conductance regulator. *Prap1*, proline-rich acidic protein 1. *Ltf*, lactotransferrin. CTR2, control group on 2 dpc. PG2, superovulation group on 2 dpc. CTR4, control group on 4 dpc. PG4, superovulation group on 4 dpc. **(A, B)** **p < 0.01. **(C-G)** *FDR < 0.05. **FDR < 0.01. ns, no significance. (n = 3).

The cooperative changes in steroid hormones in the ovary affect the estrus cycle and pregnancy. On 2 dpc and 4 dpc, we detected the contents of estrogen (E_2_) and progesterone (P_4_) in the circulating blood of mice, respectively. We found that E_2_ concentration was significantly increased and P_4_ concentration was significantly decreased after superovulation compared to the CTR group in the circulating blood at both time points (**Figures 4A, B**). We analyzed whether the genes with the estrogen and progesterone synthesis of 2 dpc and 4 dpc were DEGs. The key enzyme gene abundance in progesterone synthesis (*Cyp11a1*, *Star*, *Hsd3b1*) was increased. However, the key enzyme gene abundance of the progesterone conversion to estrogen (*Cyp17a1*, *Cyp19a1*, *Hsb17b1*) decreased (**Figures 4C, D**). The available evidence shows that the synthesis and secretion of ovarian hormones are abnormal in mice after superovulation, affecting embryo implantation.

Given that uterine changes during pregnancy are primarily regulated by estrogen and progesterone signaling, our study focused on examining the expression of the estrogen receptor (*Esr1*) and progesterone receptor (*Pgr*) expression in the uterus on 2 dpc and 4 dpc. Our results revealed an up-regulation of *Esr1* by superovulation only on 2 dpc, with no difference in *Pgr* abundance (**Figures 4E, F**). However, we observed increased circulating E_2_ concentration and decreased P_4_ on both 2 dpc and 4 dpc after superovulation (**Figures 4A, B**). Furthermore, we identified several significantly up-regulated genes on 4 dpc in the uterus, including the estrogen-responsive genes (**Figure 4G**). These findings suggest that superovulation may lead to increased exposure of the uterus to higher levels of estrogen, potentially interfering with the transition of the uterus to a receptive state.

### 3.4 Superovulation induced the abnormal gene expression related to endometrial remodeling

**Figure 5:**
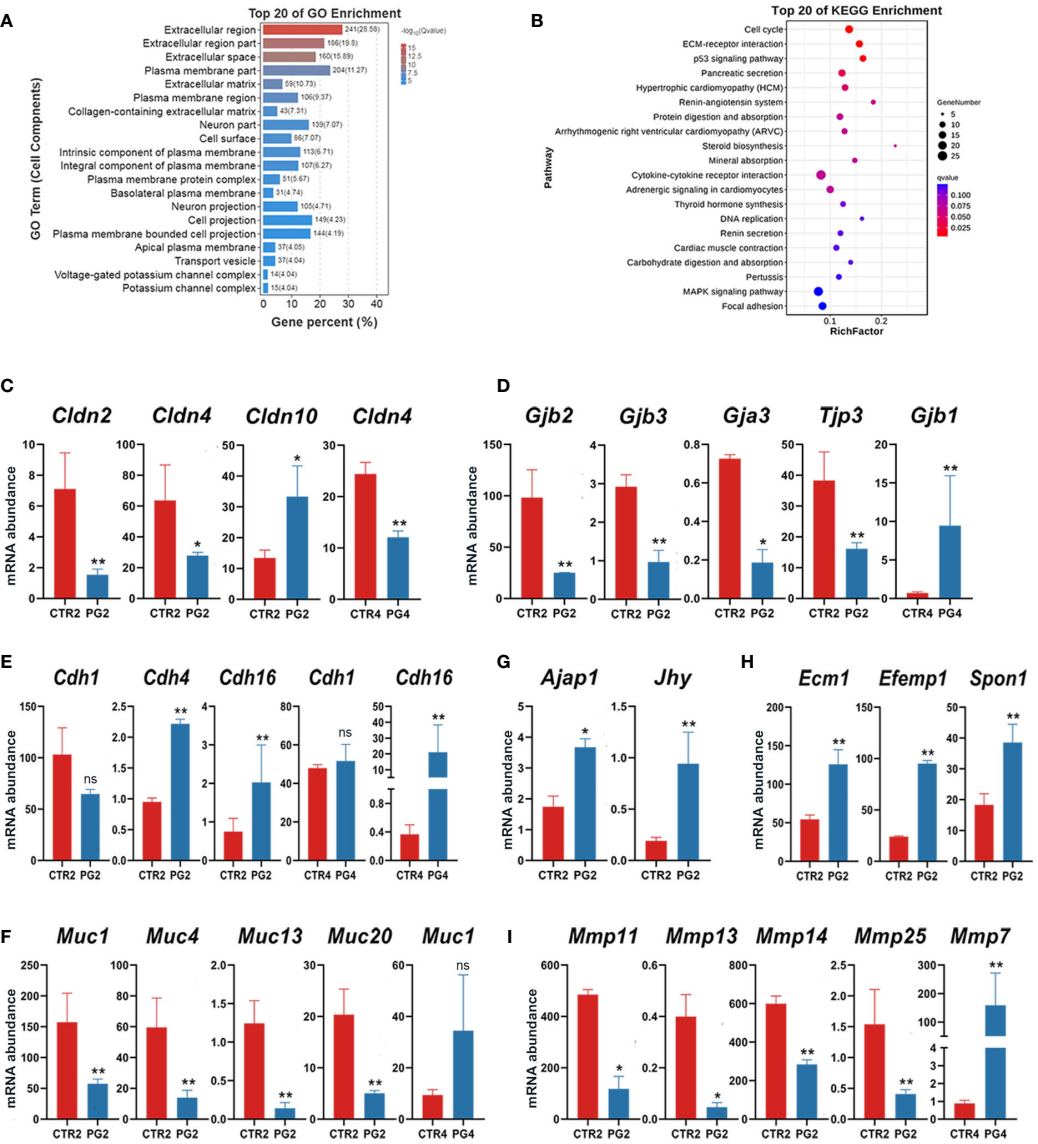
Abnormal remodeling of extracellular matrix and epithelial remodeling in the uterus after superovulation. **(A)** The top 20 of Gene Ontology (GO) analysis of differentially expressed genes (DEGs) in cell components between CTR2 and PG2 groups. **(B)** The top 20 of Kyoto Encyclopedia of Genes and Genomes (KEGG) pathway analysis of DEGs between CTR2 and PG2 groups. **(C)** DEGs of claudin (*Clnd*) family mRNA expression in CTR2 vs. PG2 groups and CTR4 vs. PG4 groups. **(D)** DEGs of gap junction protein family and tight junction protein 3 (*Tjp3*) mRNA expression in CTR2 vs. PG2 groups and CTR4 vs. PG4 groups. **(E)** DEGs of cadherin (*Cdh*) family mRNA expression in CTR2 vs. PG2 groups and CTR4 vs. PG4 groups. **(F)** DEGs of mucin (*Muc*) family mRNA expression in CTR2 vs. PG2 groups and CTR4 vs. PG4 groups. **(G)** Adherens junction-associated protein 1 (*Ajap1*) and junctional cadherin complex regulator (*Jhy*) mRNA expression in CTR2 vs. PG2 groups and CTR4 vs. PG4 groups. **(H)** DEGs of extracellular matrix component proteins mRNA expression between CTR2 and PG2 groups. **(I)** DEGs of matrix metallopeptidase (*Mmp*) family mRNA expression in CTR2 vs. PG2 groups and CTR4 vs. PG4 groups. *Gjb*, gap junction protein, beta. *Gja3*, gap junction protein, alpha 3. *Ecm1*, extracellular matrix protein 1. *Efemp1*, epidermal growth factor-containing fibulin-like extracellular matrix protein 1. *Spon1*, spondin 1, (f-spondin) extracellular matrix protein. CTR2, control group on 2 dpc. PG2, superovulation group on 2 dpc. CTR4, control group on 4 dpc. PG4, superovulation group on 4 dpc. *FDR < 0.05. **FDR < 0.01. ns, no significance. (n = 3).

An essential event during the change in uterine receptivity is endometrial remodeling. On 2 dpc, extracellular matrix-related GO terms were enriched, including the extracellular matrix (**Figure 5A**). Additionally, DEGs were significantly enriched in the cell cycle, ECM–receptor interaction, p53 signaling pathway, and pancreatic secretion (**Figure 5B**). when we screened all genes related to intercellular junctions in DEGs, we found that tight junction-related genes (*Cldn2*, *Cldn4*, *Tjp3*) and gap junction-related genes (*Gjb2*, *Gjb3*, *Gja3*) were significantly down-regulated on 2 dpc (**Figures 5C, D**), while *Cdh4*, *Cdh16*, *Ajap1*, *Jhy* were significantly up-regulated (**Figures 5E, G**). On 4 dpc, only *Cdh16* and *Gbj1* were significantly up-regulated (**Figures 5D, E**). Furthermore, four mucin family members (*Muc1*, *Muc4*, *Muc13*, *Muc20*) were found to be down-regulated on 2 dpc, with *Muc1*, which is known to be absent at implantation (**Figure 5F**). However, *Muc1* abundance increased (FDR = 0.217, *p* = 0.005) on 4 dpc, although there was no significant difference (**Figure 5F**). We also analyzed *Cdh1*, despite not being DEGs, as CDH1 deletion in endometrial epithelial cells is critical for embryo implantation. The results showed that *Cdh1* decreased (FDR = 0.183, *p* = 0.035) on 2 dpc, with no difference observed on 4 dpc (FDR = 0.999, *p* = 0.524) (**Figure 5E**). Additionally, we screened all genes about the extracellular matrix among DEGs. The results showed that the abundance of matrix structural protein genes (*Ecm1*, *Efemp1*, *Spon1*) was up-regulated on 4 dpc (**Figure 5H**), while several matrix metalloproteinase family members (*Mmp11*, *Mmp13*, *Mmp14*, *Mmp25*) were down-regulated on 4 dpc (**Figure 5I**). These results indicated that superovulation may have an impact on endometrial remodeling.

### 3.5 Superovulation led to the imbalance of the microenvironment in the uterus

**Figure 6:**
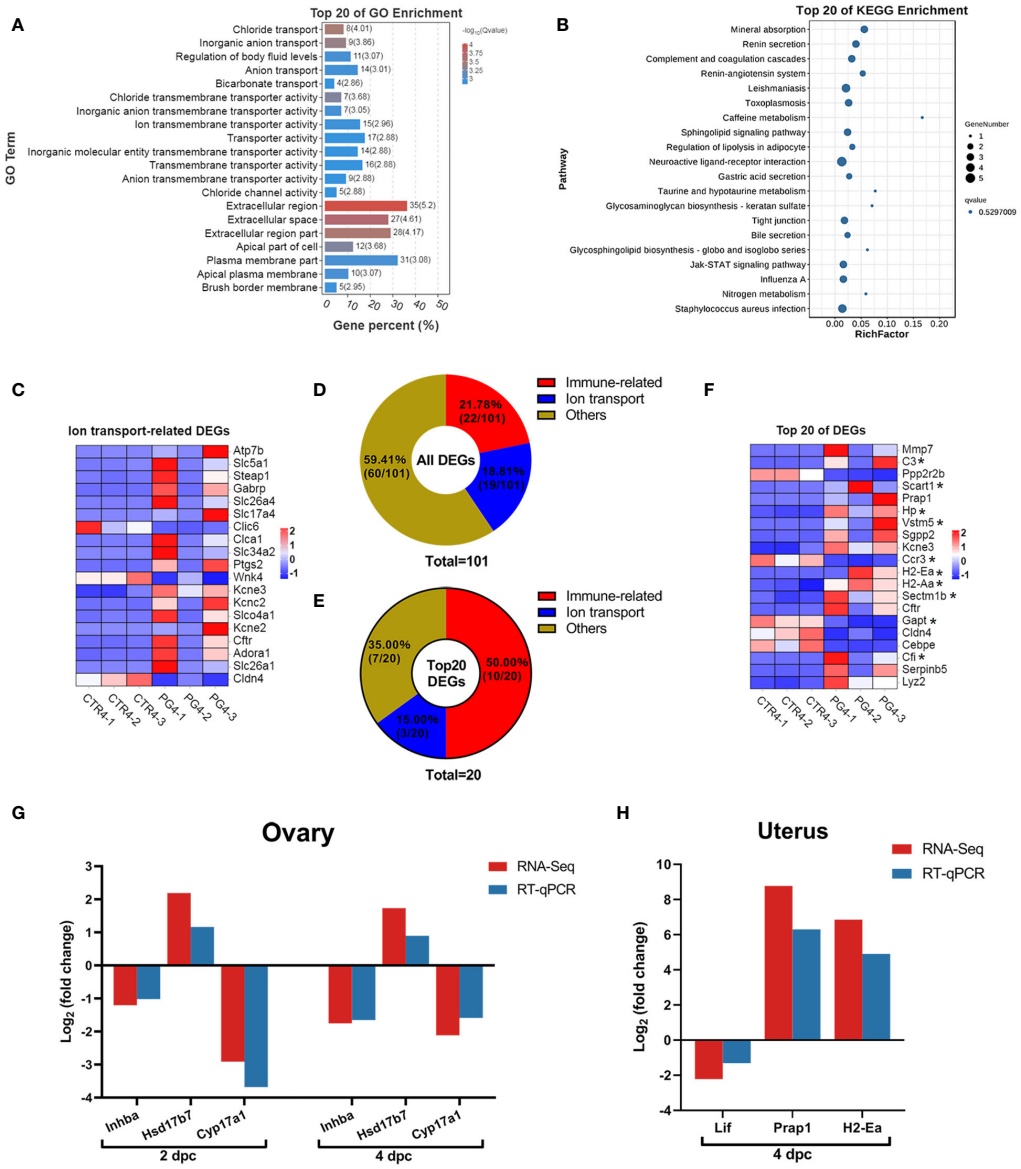
Superovulation results in abnormal ion transport and an excessive immune environment in the uterus. **(A)** The top 20 of Gene Ontology (GO) analysis of differentially expressed genes (DEGs) between CTR4 and PG4 groups. **(B)** The top 20 of Kyoto Encyclopedia of Genes and Genomes (KEGG) pathway analysis of DEGs between CTR4 and PG4 groups. **(C)** DEGs of ion transport-related gene for the heatmap between CTR4 and PG4 groups in the uterus. **(D)** All DEGs were donut-mapped between CTR4 and PG4 groups in the uterus. **(E)** The top 20 DEGs donut map between CTR4 and PG4 groups in the uterus. **(F)** The top 20 of DEGs heatmap between CTR4 and PG4 groups in the uterus. **(G, H)** The mRNA expression level of differentially expressed genes (DEGs) in the ovary and uterus was ascertained by RNA sequencing (RNA-Seq) and quantitative real-time PCR (qRT-PCR). *Immune-related genes. Each color represents the percentage of each part of the total (target genes number/total genes number) in the donut map. *Inhba*, inhibin beta-A. *Hsd17b7*, hydroxysteroid (17-beta) dehydrogenase 7. *Cyp17a1*, cytochrome P450, family 17, subfamily a, polypeptide 1. *Lif*, leukemia inhibitory factor. *Prap1*, proline-rich acidic protein 1. *H2-Ea*, histocompatibility 2, class II antigen E alpha. (n = 3).

We performed GO and KEGG enrichment analysis for DEGs in the uterus on 4 dpc. The GO analysis results showed enrichment of ion transport-related GO terms, including chloride transmembrane transporter activity, ion transmembrane transporter activity, chloride transport, and ion transport. (**Figure 6A**). The top 20 pathways enriched in the KEGG were found to be associated with mineral absorption and immune response, although no pathway showed significant enrichment (**Figure 6B**). The abnormal expression of ion channels and membrane transporters could disrupt the fluid environment in the uterus, thereby affecting endometrial function and embryo implantation. DEGs related to ion transport were significantly up-regulated on 4 dpc (**Figure 6C**), suggesting their potential role in altering the uterine fluid environment and leading to embryo implantation failure.

During pregnancy, either semen or embryos induce a wide range of immune responses. On 4 dpc, we observed that immune-related genes accounted for 21.78% of the DEGs (**Figure 6D**), and immune-related genes accounted for 50% of the top 20 DEGs (**Figure 6E**). Further evaluation of the expression of these genes revealed that the uterus had a more robust immune response after superovulation than the normal uterus during pregnancy (**Figure 6F**). These changes may contribute to the inability of the uterus to implant the embryo successfully.

### 3.6 Candidate genes identification and RNA sequencing data verification

We screened three candidate genes primarily associated with steroid hormone biosynthesis and signaling pathways in the ovary. Additionally, we focused on three candidate genes close to the implantation stage in the uterus, mainly related to immune response and signal transduction. The expression levels of these candidate genes were detected using qRT-PCR. Notably, all the qRT-PCR results exhibited the same expression pattern as the RNA-Seq results, confirming the reliability of the sequencing data (**Figures 6G, H**).

## 4 Discussion

Superovulation is a widely used technique in human reproductive medicine and animal production, but it often leads to complications such as ovarian hyperstimulation syndrome, which can impact ovarian function and embryo implantation (9, 12). Miller et al. demonstrated that GnRHa had a superovulation effect like hCG and relieved ovarian overstimulation using PMSG combined with GnRHa in a mouse model (16). Similarly, our results showed that PMSG combined with GnRHa led to an average of 30 oocytes per mouse and increased corpus luteum in the ovaries. Numerous studies have indicated that superovulation can affect the quality of oocytes and early embryos (18, 24, 25). Our study observed that mice had more blastocysts and stunted or dead embryos after superovulation than normal pregnant mice on 4 dpc, although the litter size was smaller than the number of blastocysts. Previous studies have also demonstrated that embryos implanted after superovulation were smaller than those in naturally pregnant mice, and there was a significantly higher rate of embryo reabsorption (17). However, these studies have not considered the impact of embryo quality or quantity on implantation failure after superovulation (17). In our study, we used embryo transfer to eliminate the effects of embryo number and quality and investigated the effect of superovulation on uterine receptivity. Our results indicated a decrease in embryo implantation sites after superovulation.

In mice, embryo implantation occurred between 4.5 to 5 dpc (2). To explore the effect of superovulation on embryo implantation, samples collected on the morning of the second day (2 dpc) and in the evening of the fourth day (4 dpc) after mating were selected to represent early pregnancy and the period close to implantation, respectively. We evaluated the expression of uterine receptivity-related genes (*Lif*, *Hoxa10*, and *Itgb3*) on 4 dpc. LIF is a crucial cytokine secreted by the uterine glands, playing a vital role in embryo implantation. Successful embryo implantation relies on LIF to activate the JAK-STAT pathway, leading to the phosphorylation of STAT3, which is essential for implantation (26, 27). LIF-null mice exhibit pregnancy failure (28). HOXA10 is a transcription factor that regulates the expression of factors related to embryo implantation (29). It promotes stromal cell proliferation and is involved in decidualization (29). Reduced HOXA10 contributes to implantation failure after human embryo transfer (30). Integrin is a transmembrane glycoprotein on the plasma membrane essential to embryo implantation. In pregnant mice, reduced uterine integrin expression is associated with embryo implantation failure (31). Our results indicated a significant reduction in Lif mRNA expression; however, the expression of *Hoxa10* and *Itgb3* was no significant difference. In summary, our findings suggest that the impact of superovulation on embryo implantation primarily involves affecting the uterus rather than the embryo.

To further evaluate the effect of superovulation on mouse embryo implantation, we conducted transcriptome analysis on the pre-implantation stage of both the ovary and uterus. Our findings revealed that superovulation treatment led to gene expression alterations in the ovary each time. Specifically, we observed that only 65 genes were altered between 2 dpc and 4 in normal pregnant mice, whereas superovulation resulted in a more substantial variance with 199 DEGs. Conversely, the normal uterus exhibited significant gene expression changes in pregnant mice during pre-implantation. However, only 319 differentially expressed genes were shown after superovulation, indicating that the uterus on 2 dpc was more similar to that on 4 dpc and was not yet prepared for implantation. While the ovary showed minimal gene expression changes, the uterus exhibited substantial alterations in natural pregnancy mice. Nevertheless, superovulation interferes with these changes and has the potential to lead to embryo implantation failure. These results underscore the intricate impact of superovulation on the molecular processes involved in embryo implantation.

The gonadotropin-releasing hormone (GnRH) targets the pituitary as the primary organ for follicle-stimulating hormone (FSH) and luteinizing hormone (LH) synthesis and secretion. These hormones regulate estrus, ovulation, and other biological processes (32, 33). FSH could be regulated by inhibin in the ovary (34). GO analysis indicated that DEGs were enriched in the process of FSH secretion about inhibin, with a significant decrease in the expression of inhibin-related genes on 2 dpc, suggesting that it may increase FSH secretion. Typically, FSH induces the formation of LH receptors in granulosa cells, with LH surges triggering ovulation.

Additionally, examination of the FSH and LH receptor expression in the ovary revealed reduced *Fshr* abundance and a significant increase in the *Lhcgr* abundance. In previous superovulation protocols, PMSG induced the development of many follicles to the preovulatory stage, and hCG was directly bound to LHCGR to drive ovulation (35). A Study has demonstrated that superovulation protocols triggered by GnRH and hCG can elevate *Lhcgr* expression before embryo implantation, potentially impairing ovarian function (17). Unlike hCG directly binding to LHCGR, GnRHa stimulates gonadotropin secretion, promoting LHCGR expression more akin to the natural physiological cycle. It is well known that a low pregnancy rate after superovulation is linked to abnormal expression of estrogen and progesterone (36). Our results showed that GnRHa-triggered superovulation protocols consistently showed high estrogen and low progesterone expression. Genes involved in ovarian steroid hormone synthesis exhibited an increased abundance of crucial enzyme genes for progesterone synthesis and a decreased abundance of genes involved in converting progesterone to estrogens. These changes may be attributed to negative feedback regulation from abnormal estrogen and progesterone levels. Interestingly, hCG-triggered ovulation led to the elevated expression of estrogen and progesterone levels (37). However, GnRHa-triggered ovulation resulted in decreased progesterone levels during pre-implantation.

The sustained action of LH is essential for maintaining luteal function (38). Prolonged stimulation with hCG, which directly binds to LHCGR, leads to elevated progesterone levels (39, 40). As a result, hCG is commonly used as a supplement to support corpus luteum during pregnancy (41, 42). Research has indicated that the duration of LH stimulation triggered by GnRHa is shorter than the physiological state, which may result in insufficient luteal function and even premature dissolution of the corpus luteum (43). In the human fresh embryo transfer cycle, ovulation triggered by GnRHa has demonstrated a higher rate of early miscarriage and a lower rate of live birth compared to hCG triggered (6). In a mouse model, GnRHa-triggered ovulation resulted in smaller embryos and placentas than naturally mated embryos, with significantly higher embryo resorption in GnRHa-triggered mice than in naturally pregnant mice (17). Furthermore, rabbits receiving the GnRHa trigger exhibited a high abortion rate and persistently low P_4_ concentration (44). GnRHa administration on day 7 after breeding in llamas, but there was no difference in P_4_ concentration compared to individuals with a single corpus luteum (45). These changes may be attributed to insufficient luteal function. In present studies, the results regarding progesterone levels after GnRHa-triggered ovulation varied (6, 17), but estrogen expression remained consistently high. This evidence suggests that superovulation triggered by GnRHa may lead to abnormal hormone synthesis and gonadotropin receptor expression in the ovary, potentially hindering embryo implantation.

During embryo implantation, the uterus undergoes molecular and histological changes, including inhibiting proliferation, remodeling endometrial epithelial cells, and transforming stromal cells into decidual cells. These coordinated changes in estrogen, progesterone, and certain cytokines are primarily responsible for these alterations (3, 46, 47). In ovariectomized mice, estrogen and progesterone have been shown to induce decidualization, indicating the critical role of these hormones in establishing uterine receptivity (48). Our findings revealed abnormal expression of estrogen and progesterone following superovulation. It was further confirmed that *Esr1* abundance in the uterus of the PG group was up-regulated on 2 dpc and showed no significant difference on 4 dpc compared to the CTR group. *Pgr* abundance showed no significant difference between the two groups in either 2 dpc or 4 dpc. Our results suggest that the uterus responded to high estrogen signals, as evidenced by abnormally elevated levels of genes activated by estrogen in the uterus after superovulation, even in the absence of protein level validation. Intriguingly, a high estrogen level typically leads to increased *Lif* expression (49); however, our results demonstrated that the *Lif* expression was down-regulated in the superovulation group. Those results suggest that estrogen may have a complex regulatory network for *Lif*, although the specific mechanisms require further exploration.

Endometrial remodeling is a crucial process for preparing the uterus to receive embryo implantation, involving coordinated changes in intercellular junctions, extracellular matrix remodeling, and the loss of epithelial apical-base polarity (50, 51). In this study, GO and KEGG analyses revealed that terms related to extracellular matrix remodeling were mainly enriched on 2 dpc. Further analysis indicated the up-regulation of multiple mucin family members and the down-regulation of genes associated with gap junction and tight junction. However, extracellular matrix components were up-regulated, while multiple matrix metalloproteinase family members showed down-regulation. Mucin 1 (MUC1), a member of the mucin family, plays a critical role in embryo implantation (52). MUC1 expression in epithelial cells in mice begins to down-regulate on 3.5-4 dpc, with minimal expression at the time of embryo implantation (53, 54). Our results demonstrated the down-regulation of Muc1 on 2 dpc, and a similar expression trend was observed for three other mucin family members. The result indicates that intercellular junctions and extracellular matrix remodeling are not synchronized and fail to support endometrial remodeling. E-cadherin (CDH1) is expressed at the apex of epithelial cells, and its loss during implantation indicates the loss of the epithelial cell polarity required for embryo implantation (2). Our results indicated that *Cdh1* was down-regulated on 2 dpc after superovulation, although there was no difference on 4 dpc, suggesting that premature CDH1 loss could also contribute to abnormal endometrial remodeling.

The maximum receptive period of the endometrium for the embryo is known as the implantation window (1). In mice, the implantation window typically opens on 4.5-5 dpc and lasts approximately 24 h. During this period, the embryo undergoes positioning, adhesion, and invasion, ultimately completing the implantation process (1, 55, 56). Even minor alterations in the uterine microenvironment during the implantation window can disrupt the process of embryo implantation. At the time of embryo implantation, there is a reduction in uterine cavity fluid, closure of the uterine cavity, and other changes that promote embryo attachment to the endometrium (57). Studies have shown that after superovulation, mice experience abnormal uterine fluid secretion and absorption during the implantation stage (23). GO analysis revealed significant enrichment of molecular functions related to ion transport, with many ion transport-related genes being up-regulated in the uterus on 4 dpc, including the cystic fibrosis transmembrane regulator (*Cftr*). CFTR is a chloride channel associated with uterine receptivity (58). Elevated CFTR expression during embryo implantation can lead to fluid accumulation in the uterine cavity, potentially resulting in implantation failure (57). In addition, research has indicated that increased estrogen levels induced by superovulation can lead to elevated CFTR expression and increased endometrial apoptosis.

The findings of this study indicate that superovulation can lead to changes in ion transport-related genes, possibly resulting in alterations in the uterine fluid environment that can impair embryo implantation. The immune environment of the uterus is also critical for successful implantation, as the embryo is initially perceived as a foreign body, triggering an immune response (59). However, this excessive immune response can lead to implantation failure (60–62). Our results revealed that DEGs were primarily enriched in immune-related pathways in the 4 dpc uterus. Immune-related genes were observed in up to 40% of the top 20 differential genes, with high expression levels in the superovulation group. These findings suggest that superovulation may result in excessive uterine immune defense, ultimately leading to embryo implantation failure.

## 5 Conclusions

In summary, our study has confirmed the detrimental impact of superovulation on embryo implantation through embryo transfer. Superovulation induces excessive ovarian production and prolonged presence of supraphysiological levels of estrogen. Through comprehensive transcriptome sequencing analysis, we have identified several factors contributing to implantation failure in mice following superovulation. These include abnormal gene expression related to endometrial remodeling, disrupted uterine cavity closure due to intrauterine fluid transport-related gene changes, and an intensified immune-related gene, all linked to elevated estrogen levels. As a result, future efforts aimed at mitigating the adverse effects of superovulation on pregnancy establishment should prioritize strategies to improve ovarian function and reduce the duration of elevated estrogen levels.

## Data availability statement

The original contributions presented in the study are publicly available. This data can be found here: https://www.ncbi.nlm.nih.gov/bioproject/?term=PRJNA1064015.

## Ethics statement

The animal study was approved by The Ethical Committee of Hebei Agricultural University. The study was conducted in accordance with the local legislation and institutional requirements.

## Author contributions

ML: Conceptualization, Data curation, Methodology, Writing – original draft. JH: Conceptualization, Writing – original draft, Writing – review & editing. NY: Methodology, Writing – review & editing. XL: Writing – review & editing. XW: Conceptualization, Data curation, Project administration, Writing – original draft, Writing – review & editing.
